# Measuring Labor Input: Construction Activity Counting Using IMU on Hand Tools

**DOI:** 10.3390/s23239420

**Published:** 2023-11-26

**Authors:** Xincong Yang, Yantao Yu, Heng Li, Martin Skitmore, Min-Koo Kim, Runhao Guo

**Affiliations:** 1 School of Civil and Environmental Engineering, Harbin Institute of Technology, Shenzhen 518055, China; yangxincong@hit.edu.cn; 2 Department of Building and Real Estate, The Hong Kong Polytechnic University, Hong Kong; bshengli@polyu.edu.hk; 3 Department of Civil and Environmental Engineering, The Hong Kong University of Science and Technology, Hong Kong; ceyantao@ust.hk; 4 Faculty of Society and Design, Bond University, Robina, QLD 4226, Australia; mskitmor@bond.edu.au; 5 Department of Architectural Engineering, Chungbuk National University, Cheongju 28644, Chungbuk, Republic of Korea; joekim@chungbuk.ac.kr

**Keywords:** labor input measurement, construction hand tools, IMU sensor, activity repetition counting, construction control and management, quantitative analysis

## Abstract

Efficient measurement of labor input is a critical aspect of on-site control and management in construction projects, as labor input serves as the primary and direct determinant of project outcomes. However, conventional manual inspection methods are off-line, tedious, and fail to capture their effectiveness. To address this issue, this research presents a novel method that leverages Inertial Measurement Unit (IMU) sensors attached to hand tools during construction activities to measure labor input in a timely and precise manner. This approach encompasses three steps: temporal–spatial feature extraction, self-similarity matrix calculation, and local specific structure identification. The underlying principle is based on the hypothesis that repetitive use data from hand tools can be systematically collected, analyzed, and converted into quantitative measures of labor input by the automatic recognition of repetition patterns. To validate this concept and assess its feasibility for general construction activities, we developed a preliminary prototype and conducted a pilot study focusing on rotation counting for a screw-connection task. A comparative analysis between the ground truth and the predicted results obtained from the experiments demonstrates the effectiveness and efficiency of measuring labor input using IMU sensors on hand tools, with a relative error of less than 5%. To minimize the measurement error, further work is currently underway for accurate activity segmentation and fast feature extraction, enabling deeper insights into on-site construction behaviors.

## 1. Introduction

Labor input is one of the significant indicators to evaluate construction performance. The real-time measurement and control of labor input enables the timely detection of derivations and delays between the as-built building components and the as-planned schedule. In 2008, Hong Kong local media found that contractors of the Shatin to Central Link expansion project had cut steel bars short instead of screwing them correctly into the couplers with an adequate amount of rotation [1]. Therefore, although the rebars seemed to be connected tightly in that only a few threads were exposed outside, the compression of the parts to be held together had been seriously weakened. Public concerns over this raised an important but complex issue of knowing the actual overlapping length of the connecting rebars.

Present-day methods do not provide sufficient solutions to address this problem since current project monitoring and management always focus on the outcome rather than the process. Only a few studies measure the labor input automatically and intelligently, albeit construction is a typical labor-intensive industry in which a variety of construction assignments are accomplished manually (e.g., wood formwork, reinforcement bar placement, and pipeline installation). The lack of a feasible means for the measurement and control of labor input weakens modern construction management and causes public concern in terms of project quality and safety.

The main obstacles in the automated measurement of labor input at construction sites are privacy issues and the low resolution of construction process monitoring. Emerging advanced remote sensing technologies for site monitoring, such as surveillance camera systems and laser scanning systems, provide a holistic view of the construction site instead of a fine view of each on-site factor and are expensive. It is, therefore, difficult to identify and analyze the complicated and sometimes haphazard construction activities in an often-crowded situation with various ambient occlusions. In addition, tracking workers is not possible in many countries and regions due to privacy regulations. These reasons prevent the application of advanced technologies for the automated measurement of labor input to attain a higher level of construction control and management.

To improve productivity, as well as ensure safety, a range of hand tools has been developed and implemented at construction sites, and, with the advances made in the development of construction tools, a large proportion of construction tasks are performed more quickly and accurately with less manpower [2,3]. As we move into an era of “connected things” and artificial intelligence, it is natural to think of the opportunities that new and automated techniques can provide to improve conventional process tracking ways that are open to leveraging smart tools, including concrete vibrators [4], portable power tools [5], and wire-tying hand tools [6]. This study pursues the notion that tool data will become the basis for new insights into construction processes with high resolution and proposes an Inertial Measurement Unit (IMU)-based tool data acquisition system to enable the use of hand tools to be tracked and recorded. By comparison with the correct way of using hand tools, the novel system could help control and manage the construction process in a timely manner by measuring labor input automatically and non-intrusively.

The remainder of this paper consists of five further parts. Section 2 reviews the developments of data collection methods at construction sites; Section 3 elaborates on the proposed method of automated measurement of labor input; Section 4 introduces rapid prototyping systems; and Section 5 describes a pilot study of a screw-connection task to demonstrate and validate the proposed method and system. The final section summarizes the work, its limitations, and prospects for future work.

## 2. Data Collection at the Construction Sites

The ability to identify and track construction processes effectively is a key factor for construction project success, and many studies have been made of the potential use of various advanced technologies to help managers make corrective decisions and take corrective actions in a timely manner. As listed in Table 1, in early times, the labor-intensive and tedious approach of making manual inspections and keeping paper-based records was the only way to track and manage on-site activities [7]. Then, the advent of information technology introduced the possibility of automating such tasks. The barcode identification system was first used in the construction sector in the prefabrication of concrete structures in the same way as it was applied in manufacturing to improve delivery process control [8]. Although barcoding tags were cheap, portable, and easy to read by mobile devices, they were unreliable on construction sites as the tags were vulnerable and their data capacity limited.

To enhance the reliability of automated process tracking systems in the construction industry, radio frequency (RF) transmit technology was widely adopted and rapidly developed [7,9,10]. The primary applications were mainly deployed to improve data collection efficiency at the job site entrance and storage yard. When the prefabricated units and construction materials arrived, their associated data were collected by the RF receiver and automatically saved in a database [11]. Subsequently, active, passive, and hybrid RFID tags were developed, which had a larger range of action and could carry more information, and their prices decreased. RFID, therefore, was implemented in more scenarios and facilitated on-site safety, quality inspections, and supply chain management. For example, passive RFID was attached to personal protective equipment (PPE) to check safety compliance for the awareness of users [12,13], and mobile RFID tags were integrated with the maneuver systems of hydraulic excavators and cranes to detect and prevent collision accidents [14], and stable RFID tags were even embedded in construction structures for life-cycle management and control [15]. However, although RFID technology was low-cost and easy to handle, its accuracy and robustness were insufficient for specific applications on severe construction sites because of the strength attenuation in construction materials during signal propagation [16]. As a result, ultra-wideband (UWB) and ultra-high frequency (UHF) were developed for a larger communication capability and further communication distance.

These new advanced technologies have recently been applied in localizing construction resources and navigating for the operators of heavy equipment, particularly for indoor construction projects [17]. However, both barcoding and RF transmitting technologies require readers, anchors, and routers to extract information from the tags on construction items—a situation not applicable to satellite-based GPS, another spatial technology [18]. Hence, the long-time use of GPS in outdoor positioning and navigation and its being widely accepted for deriving the identification and location information of various construction resources.

More recently, micro-electromechanical systems (MEMS) in rapid development have enabled tiny integrated devices with multiple sensors. Apart from temporal–spatial data, environmental information (e.g., temperature, humidity, and sound) can be obtained from distributed sensors, and the physiological status of individuals can be identified by wearable devices to enhance quality, safety, and overall project performance. However, whichever kind of sensors are used, data collection demands direct or close contact, and therefore, the intrusions in general on-site construction activities mean that sensor-based data collection might not be a suitable tracking tool for labor input measurement.

Another form of data collection for on-site construction is computer vision (CV) technology, which is non-intrusive and information-rich. As one of the most common instruments deployed on-site, surveillance cameras can monitor almost all the items that affect construction progress at two levels: object tracking and activity tracking [19,20]. In contrast with barcode and RFID solutions, imaging technologies capture the position, posture, and environment data simultaneously by video cameras or laser scanners. Here, object tracking aims to identify workers and recognize and locate construction resources to provide automated work progress assessment [21,22] and compare as-built and as-plan models to ensure the actual construction conforms with regulations and schedules [23]. Point cloud data directly provided by laser scanners or generated from range images or videos was used as the technology developed, enabling the percentage completion of each building component to be detected for project tracking [24,25,26,27], with activity tracking focusing on deepening and widening the scale of human motion and equipment posture detection [28,29]. As the construction industry is traditionally labor-intensive, monitoring human behaviors enables proactive project tracking so that corrective actions can be taken when abnormal construction activities are identified—potential defects being prevented in a timely manner [30,31]. Such a proactive process management method not only provides a reliable data source involving multiple construction items to boost safety and quality inspections but also implies the contribution made by each individual to the success of a project.

However, although computer vision technology has several advantages for on-site monitoring, neither the cameras nor laser scanners can capture the invisible construction processes that occur when activities or products are out of view or behind walls [32,33]. This problem is more severe in harsh construction environments. In addition, privacy issues also prevent the further use and development of CV-based on-site applications, particularly for the measurement of labor input.

To close the gap between process control requirements and efficient measurement tools, this study develops a novel method for the quantitative and timely measurement of labor input. The crucial aspect is to track and record the entire use process of hand tools to reveal the exact efforts made to achieve task goals during daily construction activities. The concept is simple but can be conducted reliably and non-intrusively without privacy issues. The theory and workflow are elaborated in what follows.

## 3. Proposed Measurement of Labor Input

**Figure 1:**
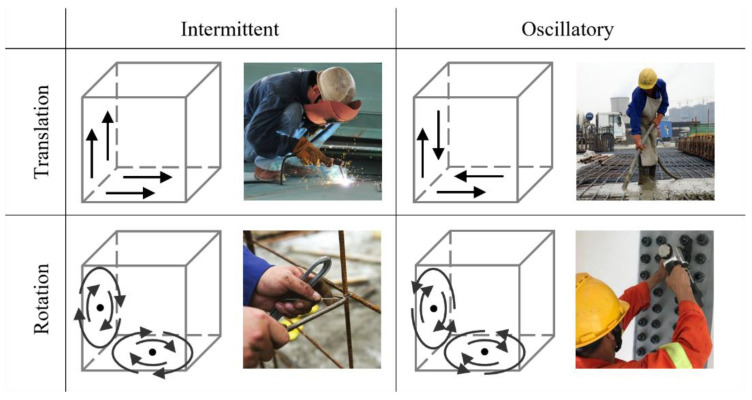
Four repetition patterns and samples of hand tools based on motion type and continuity.

Hand tools are a ubiquitous feature of construction sites, being used in such daily activities as connecting rebars and couplers using wrenches, knocking nails onto boards using hammers, and vibrating concrete for consolidation using vibrators. Most of the activities involved in their use are repetitive [34,35]. Owing to the area-restricted nature of on-site construction activities, most building products are also built up by shifting different worker trades [36,37]. Therefore, although construction activities may be haphazard because of the complicated environment and different personalities involved, it is assumed that most construction activities involving the use of hand tools are well-localized (static) and strongly periodic (stationary) [38]. Under this assumption, the repetition patterns of hand tools can be divided into four patterns by motion type and motion continuity, as shown in Figure 1.

In 3D Cartesian coordination, construction workflow using a hand tool is denoted by a vector ***P***_*t*_ = [x_*t*_, y_*t*_, z_*t*_, 1], which can be calculated by rigid transformation in homogeneous form

(1) $$P_{t+1}={H_{t}R}_{t}P_{t}$$

(2) $$\left[\begin{array}{c} x_{t+1}\\ y_{t+1}\\ z_{t+1}\\ 1 \end{array}\right]=\left[\begin{array}{cccc} 1 & 0 & 0 & h_{x}\\ 0 & 1 & 0 & h_{y}\\ 0 & 0 & 1 & h_{z}\\ 0 & 0 & 0 & 1 \end{array}\right]\left[\begin{array}{cccc} r_{11} & r_{12} & r_{13} & 0\\ r_{21} & r_{22} & r_{23} & 0\\ r_{31} & r_{32} & r_{33} & 0\\ 0 & 0 & 0 & 1 \end{array}\right]\left[\begin{array}{c} x_{t}\\ y_{t}\\ z_{t}\\ 1 \end{array}\right]$$

where *h* refers to the displacement at a time interval, and *r* represents the integrated effect components along principal axes. The basic motion pattern of translation denotes a hand tool is translating within a plane when used, such as painting, vibrating, welding, etc., which can be identified by

(3) $$H_{t}\neq I$$

(4) $$R_{t}=I$$

The other basic motion pattern of rotation denotes a hand tool rotating around a fixed axis when used. For example, an adjustable wrench, clay hammer, and rebar cutter rotate around the head, the pivot point, and the end of the handle, respectively, whereas a rebar tier rotates around the shaft axis of the handle. Rotation can be expressed by

(5) $$H_{t}=I$$

(6) $$R_{t}\neq I$$

For the motion continuity, the fundamental periodicity is represented by ${\Delta P}_{t}={\Delta P}_{t+T}$, where T is the period over time, and Δx is the deviation in a workspace. *Intermittent* continuity can be represented by

(7) $${\Delta P}_{t}=\Delta P_{t+T/2}=\Delta P_{t+T}$$

Meanwhile, oscillatory continuity is described by

(8) $${\Delta P}_{t}=-\Delta P_{t+T/2}=\Delta P_{t+T}$$

In practice, stationary repetitive activities are relatively rare, and there may be a mixture of repetition patterns due to the complicated task and the harsh environment involved. The present study, therefore, considers the periodic motion to be near-stationary, which makes the proposed method more robust and general.

**Figure 2:**
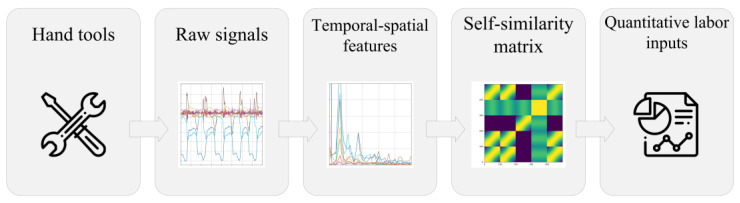
Overview of the proposed method for labor input measurement.

Based on the stationary repetition assumption, a 3-step repetition estimation process is proposed: first, various signals are collected from on-site activities and converted into suitable temporal–spatial feature sequences for data augmentation; second, each element is compared with other elements in the sequences to generate a temporal self-similarity matrix; finally, path and block identification is carried out to segment the sequences and count the repetitions for labor input measurement as shown in Figure 2. Each step is elaborated here in the next three sections.

### 3.1. Temporal–Spatial Feature Extraction

Raw signals collected from hand tools are always mixed with noises. These noises are the results of the harsh environment, different personal work habits, and instrument errors during deployment and measurement. To alleviate their negative influences, the various temporal–spatial features are extracted from the raw data of the IMU sensors to generate effective feature sequences. Table 2 lists the appropriate features verified in previous research and many applications. Notably, the features in the time domain are not only extracted from data along an independent axis, but they also contain coefficients and covariances from each pair and the magnitude of three axes in the Cartesian coordinate system.

### 3.2. Self-Similarity Matrix

After feature extraction, a series of hand tool data are divided into several fixed-length feature sequences. Let x be the feature sequence in the feature space $R^{1\times N}$. The concept of the self-similarity matrix (SSM) is to compare each sequence in F with the other sequences, resulting in a square matrix $S\in R^{M\times M}$. The SSM element is defined by

(9) $$S_{i,j}≔s(x_{i},x_{j})$$

where $x_{i},x_{j}\in F$ for i,j∈[1:M] and $s\left(.\right)$ is the similarity function that calculates a score measuring the similarities between vectors. A simple but efficient similarity function is the inner product of latent vectors. To locate the value of similarity measurements in the interval [−1,1], all feature vectors are normalized with respect to the Euclidean norm; thus, the SSM diagonal elements are obviously largest at 1, and recurring patterns among feature sequences could be highlighted in the form of structures with larger similarity values. Since the feature sequences are denoted by matrix representation $X\in R^{M\times N}$, the SSM can, therefore, be calculated by

(10) $$S=XX^{T}$$

### 3.3. Path and Block Identification

With respect to repetition patterns, the most prominent structures are paths and blocks. The basic idea is that repeating feature sequences from different repetitions but corresponding to the same activity segmentation results in paths or slashes along the SSM main diagonal so that the repetitive attributes account for path-like structures. Meanwhile, each feature sequence is similar to all the other feature vectors within a repetitive action, leading to a block with larger elements in the SSM, which means they have homogeneity properties.

Drawing from the dynamic time warping (DTW) algorithm for alignment, a path P of length L is defined by

(11) $$P=(\left(n_{1},m_{1}\right),\ldots ,\left(n_{l},m_{l}\right),\ldots ,(n_{L},m_{L}))$$

where $n_{l}\in [1:N]$ and $m_{l}\in [1:M]$ for l∈[1:L] with the monotonicity condition $n_{1}\leq n_{2}\leq \ldots \leq n_{L}$ and $m_{1}\leq m_{2}\leq \ldots \leq m_{L}$. Also, the step size $\left(n_{l+1},m_{l+1}\right)-\left(n_{l},m_{l}\right)$ should satisfy the continuity condition to avoid replications and omissions. For each path, a score is given to evaluate the entire quality of two similar feature sequences by

(12) $$Q\left(P\right)=\sum_{l=1}^{L}s(n_{l},m_{l})$$

Similar to the path, a block of width W and height H is defined by

(13) $$B=(\left(n_{1},m_{1}\right),\ldots ,\left(n_{w},m_{h}\right),\ldots ,(n_{W},m_{H}))$$

The corresponding score of the block is defined by

(14) $$Q\left(B\right)=\sum_{w=1}^{W}\sum_{h=1}^{H}s(n_{w},m_{h})$$

**Figure 3:**
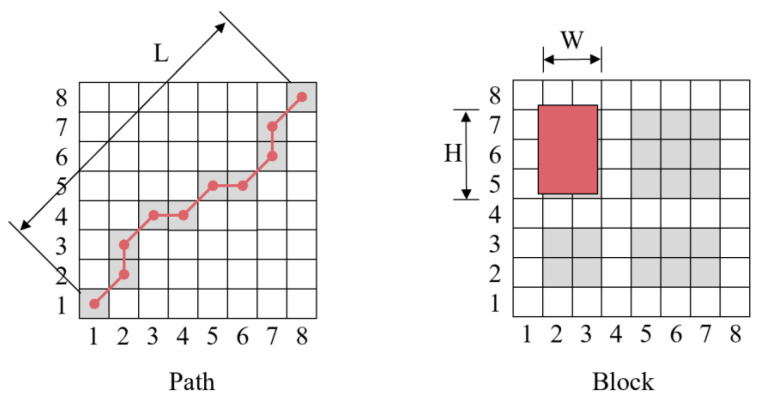
Path and block in self-similarity matrix.

As shown in Figure 3, similar structures of repetition patterns are identified based on the SSM paths and blocks. Given a path P with a high score, it is concluded that the associated segments N and M are path-like. Similarly, if a block B with a high score is identified, segments N and M are block-like.

The repetitive segments always perform a similar SSM structure; repetition counting could, therefore, be induced by calculating and counting the SSM paths and blocks.

## 4. IMU-Based Tool Data Acquisition System

To verify the proposed novel approach for labor input measurement, we developed a technical prototype to collect the movements of hand tools during construction activities. The entire system is composed of perception, network, and application layers.

### 4.1. Perception Layer

**Figure 4:**
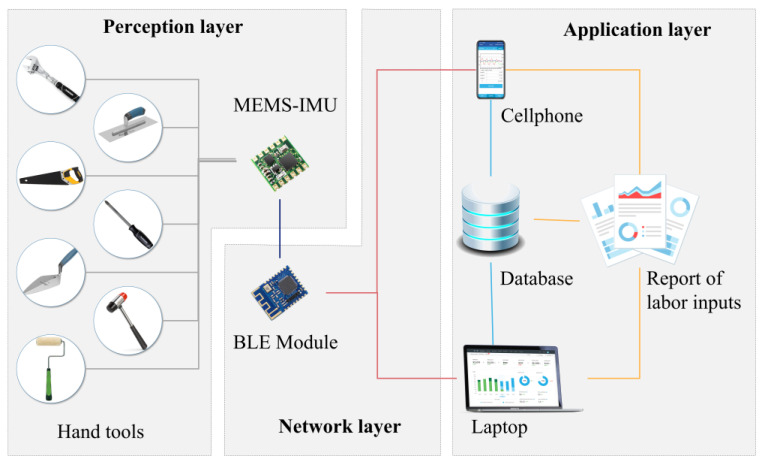
Architecture of the proposed system.

As shown in Figure 4, the physical device to collect data from hand tools is a micro-electromechanical system (MEMS) consisting of an Inertial Measurement Unit (IMU), a microprocessor, and a Bluetooth low energy (BLE) module. The IMU sensor (produced by WIT-Motion^®^, WitMotion Shenzhen Co., Ltd., Shenzhen, China) is made of a tri-axis gyroscope, tri-axis accelerometer, and tri-axis magnetometer, along with a thermometer, enabling the measurement of angular velocity, acceleration, local magnetic field, and temperature. The tiny chip is small, tight, and portable enough to be attached to the handle of a hand tool or embedded in the motherboard of a power hand tool.

### 4.2. Network Layer

Since a larger number of hand tools are used on-site, the Bluetooth Mesh is applied to establish many-to-many communications. A conventional BLE module can only transit signals to less than 7 visualization terminals, while the BLE supporting Bluetooth Mesh allows more than 50,000 transitions in the wireless network [39].

### 4.3. Application Layer

**Figure 5:**
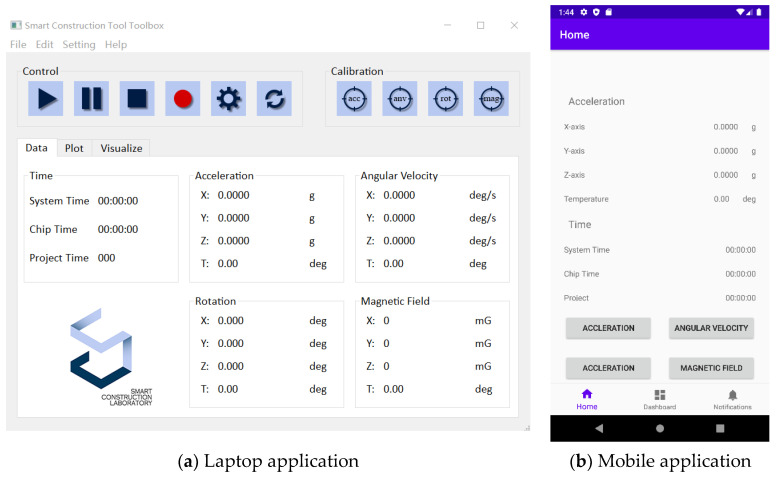
User interface of the IMU-based hand tool data acquisition system.

Cell phones and laptops with BLE modules are deployed to process and visualize signals collected from hand tools, receive signals, analyze data by the proposed approach, and generate reports for quantitative and objective labor input measurement. In addition, these terminals also send the outputs to a cloud database for historical backup. As Figure 5 shows, the computer application is built with the Qt Designer 6.2 and Python 3.8 programming language, while the mobile application is developed with the Java programming language.

## 5. Pilot Study—Rotation Counting Test for Screw Connection

Screw connections are widely used at construction sites for pipeline installation, reinforced concrete, and steel structures. However, in the modern construction industry, the screw connections are always hidden for aesthetic and safety reasons. If the connections do not have the correct torque and are not reliable, serious accidents may occur, as in the case of faulty work in Hong Kong, where steel rebars were cut prior to being connected to screw couplers embedded in a concrete underground platform [1]. Hence, this study provides experiments for the screw-connection task because of its significance and invisibility.

### 5.1. Experimental Setting

To validate the proposed IMU-based tool data acquisition method and system, the screw-connection experiment was carried out in the Hong Kong Polytechnic University’s Smart Construction Lab. A total of 5 workers between 22 and 30 years old were recruited to turn a single screw connection. Prior to the task, the experiment was explained to the participants in detail, and trials without data collection were performed to check their understanding and ability.

**Figure 6:**
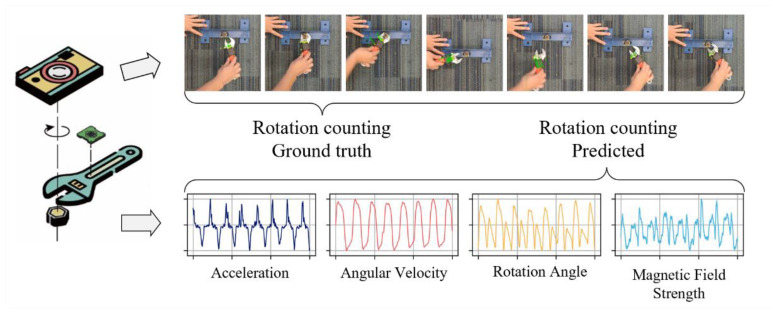
Schematic diagram of the experiments in lab.

As shown in Figure 6, the participants were required to apply a specified turn on an adjustable wrench and restrain the unturned steel structure from rotation. Once the bolt and nut were snug-tightened, the final rotated position was verified and recorded as the ground truth of the labor input measurement through a high-resolution digital video camera deployed on the top of the steel structure with 30 fps. The proposed IMU-based hand tool data acquisition system was also attached to the handle of the wrench for raw signal collection with a sampling frequency of 100 Hz. The experiment was performed under the supervision of an inspector to resolve and clarify any doubts and prevent possible injuries.

Before the experiment, the IMU sensors were calibrated to alleviate the negative noise effect from the installation and environment. To save time and cost, a robust and easy IMU calibration without external equipment was adopted. The calibration procedure required the integrated sensor to collect accelerations, angular velocities, and magnetic fields in different static positions for 10 s. For the calibration of accelerometers, average filtering was applied in each static interval, and the results were compared with the known local gravity accelerations for estimation. To calibrate the gyroscope triad, the Allan variance and orientation were calculated by integration algorithms [40]. Using the acceleration and magnetic field readings for reference, the unknown gyroscope parameters were estimated. Finally, the measurement noises of magnetometers were neglected due to the magnetic field signals being stable at static intervals.

### 5.2. Data Processing

**Figure 7:**
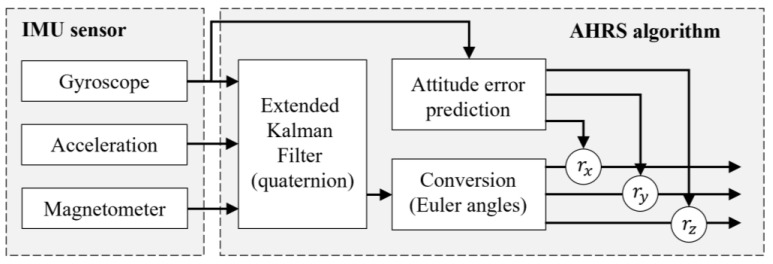
Diagram of the fusion algorithm for AHRS.

Based on the deployment of the IMU sensor on the adjustable wrench, it was clear that the screw-connection assignment was conducted with a repetitive pattern of oscillatory rotations. Since gyroscopes are resistant to vibrations but not reliable for long-term monitoring, an attitude and heading reference system (AHRS) algorithm was applied (Figure 7) to improve the accuracy of the rotation data by combining the accelerations and the local magnetic field data from the accelerometer and magnetometer, which were stable in the long term [40,41].

**Figure 8:**
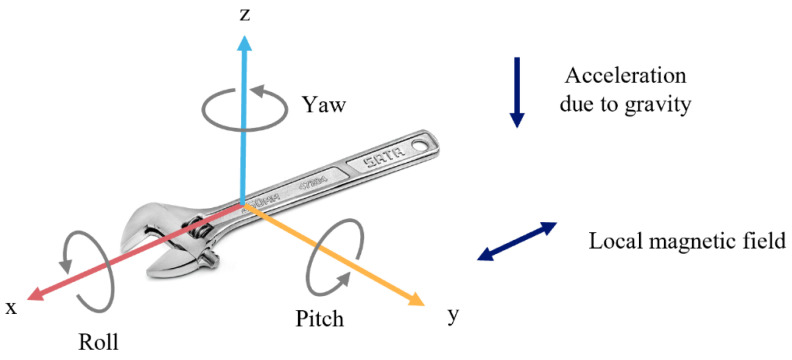
Deployment of the IMU sensor on hand tools.

As Figure 8 shows, in the Earth’s gravitational field, any axis with a value of g in the accelerometer output of a stationary item is obviously aligned with the Earth’s downward gravity force. The roll ϕ and pitch θ angles can, therefore, be evaluated by

(15) $$\phi =\tan^{-1}\frac{a_{y}}{a_{z}}$$

(16) $$\theta =\tan^{-1}\frac{-a_{x}}{\sqrt{a_{y}^{2}+a_{z}^{2}}}$$

where a is the acceleration readings from the IMU sensor. Meanwhile, in the Earth’s magnetic field, the components of the down/up and north axes are parallel to the Earth’s surface, while the component along with the east direction is empty. The yaw ψ angle can thus be calculated by

(17) $$\psi =\frac{h_{x}}{h_{y}}-\Delta \psi$$

where *h* refers to the magnetic signals from IMU and Δ*ψ* refers to the declination constant at Hong Kong, which is approximately 2°59′ relative to west.

In addition, the rotation angles could also be estimated by the integral of angular velocity from the gyroscope in IMU. Fusing these sensor data by the AHRS algorithm, the static accuracy of the rotation measurement could reach 0.5 deg/s, and the dynamic error was less than deg/s.

### 5.3. SSM Calculation and Activity Counting

**Figure 9:**
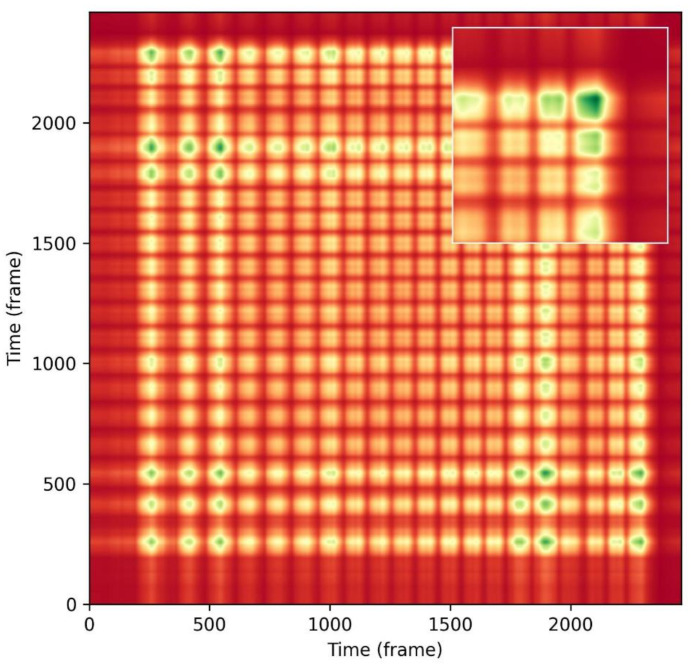
Sample of the generated self-similarity matrix.

As shown in Figure 9, the features and SSM were extracted and calculated from the collected and processed jaw angles. It can be seen that there are numerous block-like blocks along the horizontal and vertical lines in the sample SSM, exposing the repetition pattern during the use of the adjustable wrench. According to our assumption, the SSM is a symmetric matrix where the diagonal elements are the largest since each activity is always similar to itself, which is verified in this matrix plot. In addition, the SSM blocks appear at regular intervals, suggesting the screw-connection task using an adjustable wrench is oscillatory and could be monitored in a lean way.

**Figure 10:**
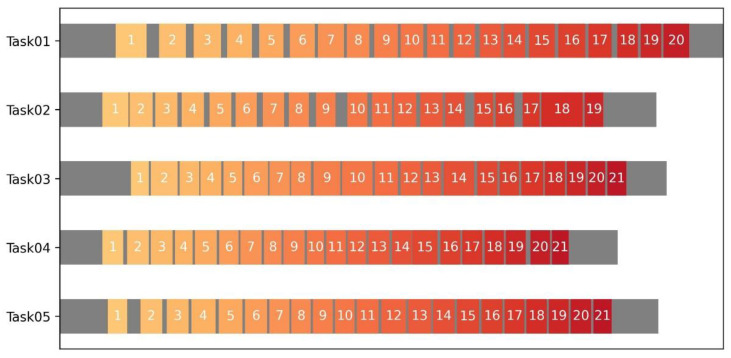
Results of the Repetition counting.

Therefore, as illustrated in Figure 10, in applying the block identification method, the number of repetitive activities is obtained as an objective and automatic measurement of labor input. In this horizontal bar chart, the gray color refers to the non-repetitive activities during the screw-connection task; meanwhile, the colors from orange to red suggest the repetition counting numbers of the rotation movement. Here, a unit repetition refers to applying torque by an adjustable wrench until the bolt or nut rotates almost 150 degrees, which was constricted by the limited workspace in this experiment. By the comparison between these tasks, Task02 seemed to be incomplete as its repetition counting of 19 was lower than that of the other tasks. In addition, the bar chart also clearly reveals the distribution of preparation work and finishing touches in construction activities, providing a quantitative perspective for labor input measurement.

Table 3 lists the results obtained from the comparison between the ground truth from videos and the identified repetition counting from the proposed method. From this, it can be concluded that the relative error of 5% is acceptable.

### 5.4. Discussion

**Figure 11:**
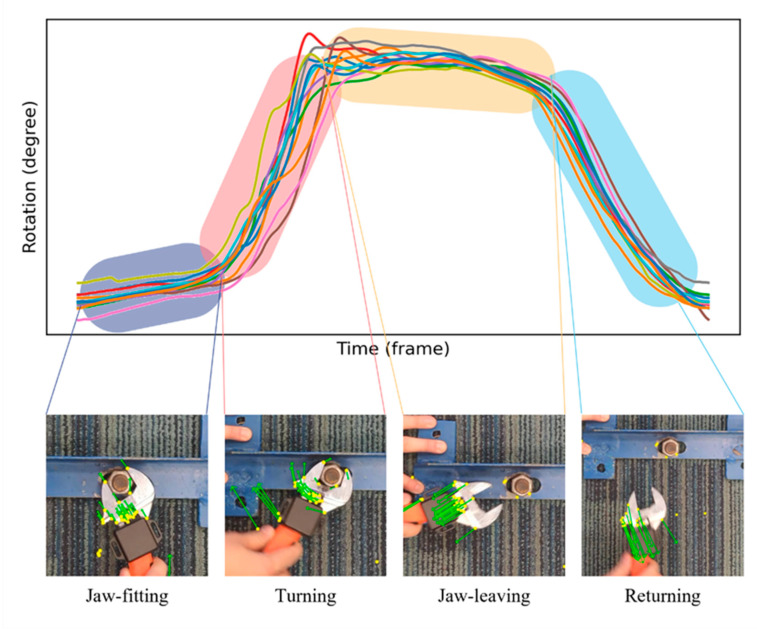
Activity segmentation of screw-connection task.

Overall, the pilot study indicates that the proposed IMU-based labor input measurement through monitoring the use of an adjustable wrench is efficient for oscillatory rotation tasks. However, there are still discrepancies between the ground truth and the prediction. To analyze this further, as shown in Figure 11, we separate the repetition unit of the screw-connection task into four phases: jaw-fitting, turning, jaw-leaving, and returning. Here, jaw-fitting refers to positioning a wrench around the nut or bolt, the turning phase is to apply torque to turn the wrench with a specific rotation, jaw-leaving literately describes the process of separating apart the wrench and bolt or nut and returning is to return to the beginning position for the next repetition. As the plot shows, these four phases account for different time intervals and perform different motion patterns, as well as different time and frequency domain features. The window size of the feature extraction, therefore, has a considerable impact on the evaluation of the proposed IMU-based method.

**Figure 12:**
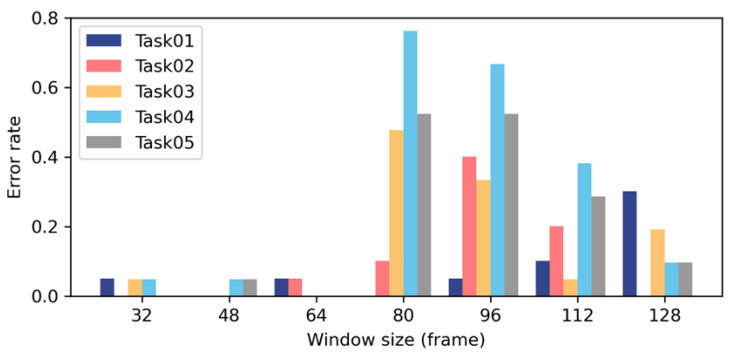
Impact of window sizes on error rate.

As Figure 12 describes, different sizes of windows were examined and compared in the pilot study. This shows that window sizes 32, 48, and 64 performed best, with the lowest average error rate. With the enlargement of window size, the error rate dramatically increases at first and decreases at the end. The possible reasons include (1) if the window size is defined as larger than the activity segmentation, different features might fuse and become complicated; and (2) the fast Fourier transform algorithms for frequency domain feature extraction only support 32, 64, 128, … samples. For other window sizes, some components of the frequency domain features might be lost.

The preliminary limitation of this study is the deviation of the repetition patterns. Here, the deviation between workers is the result of different personalities and working habits, while the deviation over time is a consequence of physical exertion at work, in terms of the repetition patterns changing because of physical and mental fatigue and exhaustion. Although deviations in repetition patterns could lead to erroneous counting, it could also be treated as an indicator for anomaly detection for construction management.

## 6. Conclusions and Future Work

The efficient measurement of labor input is of great value to the construction industry since it allows managers and workers to monitor production and quantify productivity in a timely and quantitative manner. This study proposes a novel construction activity counting method that quantifies labor input by tracking and recognizing similar motion sequences of hand tools. Accordingly, the study establishes a preliminary IMU-based tool data acquisition workflow and system, which is composed of feature extraction, self-similar matrix calculation, and local structure identification for automated repetitive activity counting. Finally, the study verifies the concept and examines the feasibility of the prototyping in a screw-connection task. The results of the pilot study show the high accuracy and effectiveness of the method for the automated labor input measurement. Therefore, such IMU sensors could be embedded in general hand tools and the method implemented in various manual activities for an objective and quantitative form of construction control and management.

The experimental work in this study is so far limited to a single activity of screw connection, and further work is needed in trialing the system with other kinds of construction tools and activities. There is also the potential for further development of deep learning techniques for end-to-end smart labor input measurement if the edge devices are in widespread use at construction sites.

## Figures and Tables

**Table 1:** Common data acquisition methods at construction sites.

Data Acquisition Method	Automation	Data Format	Data Collected
Manual inspection	Low	Paper-based	Workers, equipment, materials
Radio frequency identification (RFID)	Mediate	Datastream	Workers, precast components
Ultra-wideband (UWB)	Mediate	Datastream	Workers, equipment
Ultra-high frequency (UHF)	Mediate	Datastream	Workers, equipment
Global position system (GPS)	Mediate	Datastream	Workers, equipment
Wearable devices	Mediate	Datastream	Workers, equipment
Computer vision (CV)	High	Images/videos	Workers, equipment, materials, precast components
Laser scanning	High	Point clouds	Workers, equipment, materials, precast components

**Table 2:** Temporal–spatial features of the raw signals along an axis.

Feature Type	Feature Name	Description
Temporal	Arithmetic mean	$mean=\frac{1}{N}\sum_{i=1}^{N}x$
Temporal	Standard deviation	$std={\left(\frac{1}{N-1}\sum_{i=1}^{N}{\left(x_{i}-\overset{-}{x}\right)}^{2}\right)}^{\frac{1}{2}}$
Temporal	Median	$median(x_{i})$
Temporal	Maximum	$max(x_{i})$
Temporal	Minimum	$min(x_{i})$
Temporal	Range	$\max\left(x_{i}\right)-min(x_{i})$
Temporal	Skewness	$skew=\frac{\frac{1}{N}\sum_{i=i}^{N}{\left(x_{i}-\overset{-}{x}\right)}^{3}}{{\left(\frac{1}{N}\sum_{i=i}^{N}{\left(x_{i}-\overset{-}{x}\right)}^{2}\right)}^{\frac{3}{2}}}$
Temporal	Kurtosis	$kurt=\frac{\frac{1}{N}\sum_{i=i}^{N}{\left(x_{i}-\overset{-}{x}\right)}^{4}}{{\left(\frac{1}{N}\sum_{i=i}^{N}{\left(x_{i}-\overset{-}{x}\right)}^{2}\right)}^{2}}-3$
Temporal	Energy/Power	$\frac{1}{N}\sum_{i=1}^{N}x^{2}$
Temporal	Root Mean Square	${\left(\frac{1}{N}\sum_{i=1}^{N}x^{2}\right)}^{\frac{1}{2}}$
Temporal	Nth percentile	percentile(x,n)
Spatial	Covariance	$\frac{1}{N-1}\sum_{i=1}^{N}\left(x_{i}-\overset{-}{x}\right)\left(y_{i}-\overset{-}{y}\right)$
Spatial	Autoregression coefficient	$\frac{\sum_{i=i}^{N}\left(x_{i}-\overset{-}{x}\right)\left(y_{i}-\overset{-}{y}\right)}{{\left(\sum_{i=i}^{N}{\left(x_{i}-\overset{-}{x}\right)}^{2}\right)}^{\frac{1}{2}}{\left(\sum_{i=i}^{N}{\left(y_{i}-\overset{-}{y}\right)}^{2}\right)}^{\frac{1}{2}}}$
Frequential	Maximum frequency	$max(f_{i})$
Frequential	Weighted mean frequency	$\frac{\sum_{i=i}^{N}W_{i}f_{i}}{\sum_{i=i}^{N}W_{i}}$

**Table 3:** Repetition counting comparison between ground truth and prediction.

Task	Ground Truth	Prediction	Average Relative Error
01	20	20	0%
02	20	19	−5%
03	21	21	0%
04	21	21	0%
05	21	21	0%

## Data Availability

Data are contained within the article and Supplementary Materials.

## Supplementary Materials

The following supporting information can be downloaded at: https://www.mdpi.com/article/10.3390/s23239420/s1.
